# A chimeric mouse model to study human iPSC-derived neurons: the case of a truncating *SHANK3* mutation

**DOI:** 10.1038/s41598-020-70056-4

**Published:** 2020-08-07

**Authors:** Aline Vitrac, Stéphanie Pons, Marta Balkota, Nathalie Lemière, Célia Raïs, Jean-Pierre Bourgeois, Uwe Maskos, Thomas Bourgeron, Isabelle Cloëz-Tayarani

**Affiliations:** 1 Human Genetics and Cognitive Functions, CNRS UMR 3571 « Genes, Synapses and Cognition », Université de Paris, Institut Pasteur, Paris, France; 2 Integrative Neurobiology of Cholinergic Systems, CNRS UMR 3571 « Genes, Synapses and Cognition », Institut Pasteur, Paris, France; 3 Collège Doctoral, Sorbonne Université, Paris, France

**Keywords:** Genetics, Neuroscience, Stem cells, Diseases

## Abstract

Using human induced pluripotent stem cells (iPSC), recent studies have shown that the events underlying autism spectrum disorders (ASD) can occur during neonatal development. We previously analyzed the iPSC-derived pyramidal cortical neurons of a subset of patients with ASD carrying de novo heterozygous mutations in postsynaptic SHANK3 protein, in culture. We reported altered spinogenesis of those neurons. The transplantation of human iPSC-derived neuronal precursors into mouse brain represents a novel option for in vivo analysis of mutations affecting the human brain. In this study, we transplanted the neuronal precursor cells (NPC) into the cortex of newborn mice to analyze their integration and maturation at early stages of development and studied axonal projections of transplanted human neurons into adult mouse brain. We then co-transplanted NPC from a control individual and from a patient carrying a de novo heterozygous *SHANK3* mutation. We observed a reduction in cell soma size of selective neuronal categories and in axonal projections at 30 days post-transplantation. In contrast to previous in vitro studies, we did not observe any alteration in spinogenesis at this early age. The humanized chimeric mouse models offer the means to analyze ASD-associated mutations further and provide the opportunity to visualize phenotypes in vivo.

## Introduction

The use of human-derived cellular models to study neurodevelopmental disorders such as autism spectrum disorders (ASD) is an appropriate approach to link causal genetic alterations to molecular mechanisms that may occur in humans during pre- and postnatal brain development. The “SH3 and multiple ankyrin repeat domain 3” (SHANK3) is a member of the *SHANK* gene family located on chromosome 22 in humans which contains multiple structural domains as we previously demonstrated by homology modeling^1^. Mutations in *SHANK3 *represent some of the best-known genetic causes for ASD and account for the severe intellectual deficiency (ID) and language deficits observed in 1–2% of patients with ASD^2,3^. *SHANK3* haploinsufficiency also contributes to the clinical symptoms of patients with Phelan-McDermid syndrome which presents a deletion of chromosome 22q13 that includes *SHANK3* in the large majority of cases^4,5^. SHANK3 is a critical partner of a major signaling complex expressed at postsynaptic densities (PSD) of glutamatergic synapses which involves cytoskeletal networks at both the soma and the neurites of neuronal cells^6^. SHANK3 therefore plays a crucial role in synapse formation and dendritic spine maturation. The fundamental role of SHANK3 at such excitatory synapses was initially investigated by using genetically modified mouse models^7–9^, or by overexpressing mutated SHANK3 protein in transfected rat neurons in culture^10^. Human induced pluripotent stem cells (iPSC)-derived neurons represent a valuable model for in vitro analysis of SHANK3 deficiencies in humans^11–15^. Accordingly, we recently selected four independent patients with heterozygous truncating de novo* SHANK3* point mutations who had previously been characterized in our laboratory^1,3^. We generated the corresponding human iPSC for their selective reprogramming into cortical neurons^13,16^. We then examined the effects of *SHANK3* haploinsufficiency on the levels of *SHANK3* mRNA and on the spine morphogenesis to demonstrate the correlation between spinogenesis defects and *SHANK3* levels^1^. In accordance with earlier reported results, our data from these studies with cultured human neurons confirmed the existence of early synaptic deficits that may occur in the brain of ASD patients who carry *SHANK3* mutations. Together with local synaptic deficits, a defective short-range cortico-cortical wiring was recently reported in a mouse model presenting a homozygous loss of the Shank3B isoform^17^. The human cortex is an important region that undergoes multi-developmental processes. Recent findings from animal models suggest that pathological modifications that lead to ASD start before birth at early stages of cortical development^18^. Another recent study with iPSC, derived from ASD patients, has shown a dysregulation of specific neurodevelopmental gene modules which occurs at stages of neuronal precursors^19^. The neuronal connections that are observed in two-dimensional cultures cannot fully reflect the in vivo conditions, and this represents an important issue for interpretation of data. Recent alternative protocols including the transplantation of human cells, derived either from human embryonic stem cells (ESC) or from human iPSC, offer the possibility to analyze the projection patterns of grafted human cortical neurons within the adult mouse brain^20^. These transplantation protocols have also been used to repair brain lesions by employing mouse ESC-derived cortical neurons^21,22^. In the present study, we used a human neuronal-chimeric mouse by transplanting human NPC into the brain of immune-deficient newborn mice. We analyzed the in vivo integration and maturation of cortical cells from a control individual in different regions of mouse brain. We then co-transplanted, for the first time, the neurons from the control individual and from an ASD patient with a heterozygous truncated SHANK3 mutation to study at early stages of neuronal maturation some key morphological parameters of mutated neurons, under in vivo conditions.

## Results

### Dendritic spine morphogenesis under in vitro and in vivo conditions

**Figure 1:**
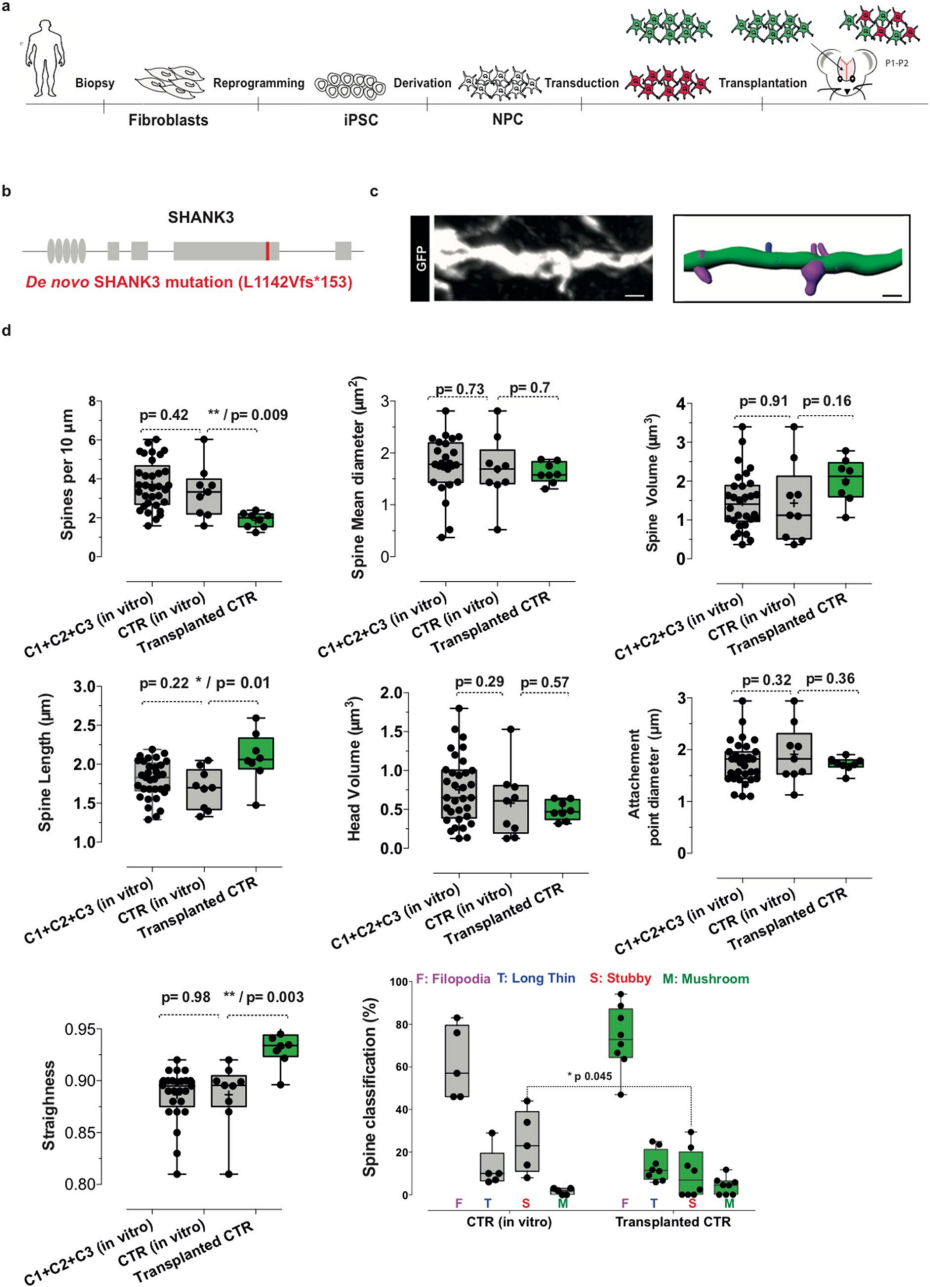
Quantitative analysis of primary dendritic spines of human iPSC-derived neurons kept either in culture or transplanted into neonate mice brain. (**a**, **b**) Schematic representation of the steps of experimental design, illustrating the production of neuronal precursor cells derived from human iPSC after the initial reprogramming from fibroblasts, the transduction of NPC with fluorescent markers (GFP and/or mCherry), and their transplantation into newborn mice brain. Using confocal microscopy, the study consists of analysis of dendritic spines together with comparative analysis of soma areas and axonal projections between co-transplanted CTR and ASD cells from a male patient with a frameshift mutation in *SHANK3* referred as L1142Vfs*153 located in the proline-rich domain of SHANK3 protein^1^. (**c**) Primary dendrite segment of human pyramidal neuron at 50 days post-transplantation with corresponding 3D reconstruction. Scale bar = 4 μm. Dendrite segment (green color) are endowed with four categories of spines: Filopodia (pink color), Long Thin (blue color), Stubby (red color) and Mushroom (green color). (**d**) Comparative quantitative analysis of spine morphological parameters using the Imaris Software as described in Materials and Methods. In vitro data are from our previous study^1^ of pyramidal neurons kept for 45–50 days in culture. In vivo data are from two independent mice perfused at 50 days post-transplantation. Number of dendrites is indicated in the graph. Data are presented as mean ± SEM. Statistical analysis was performed using a Mann–Whitney test. *p < 0.05; **p < 0.01.

The experimental design of the study as well as the illustration of spine remodeling is depicted in Fig. 1a–c. For transplantation, we used immunodeficient NOD/SCID mice to achieve full integration of cells beyond 2 weeks post-transplantation as described previously^20,23^.

We transplanted the NPC, without initial differentiation in culture, into the cortex of newborn mice and followed their maturation in vivo. We compared the morphological parameters of dendritic spines in a pool of neurons from three control individuals with the selected control neurons (CTR) which were either kept in vitro for 40–50 days or transplanted into the cortex of newborn mice for analysis at 50-day-old. Our data show a significant reduction in the density of spines as well as a decrease in the percentage of stubby spines in transplanted neurons, as compared to neurons which were kept in vitro. We also observed a significant increase in spine straightness and length in transplanted neurons, as compared to in vitro conditions (Fig. 1d). Other morphological parameters of the spines remained unchanged (Fig. 1d).

### Mapping of axonal projections of transplanted neurons and immunofluorescence studies

**Figure 2:**
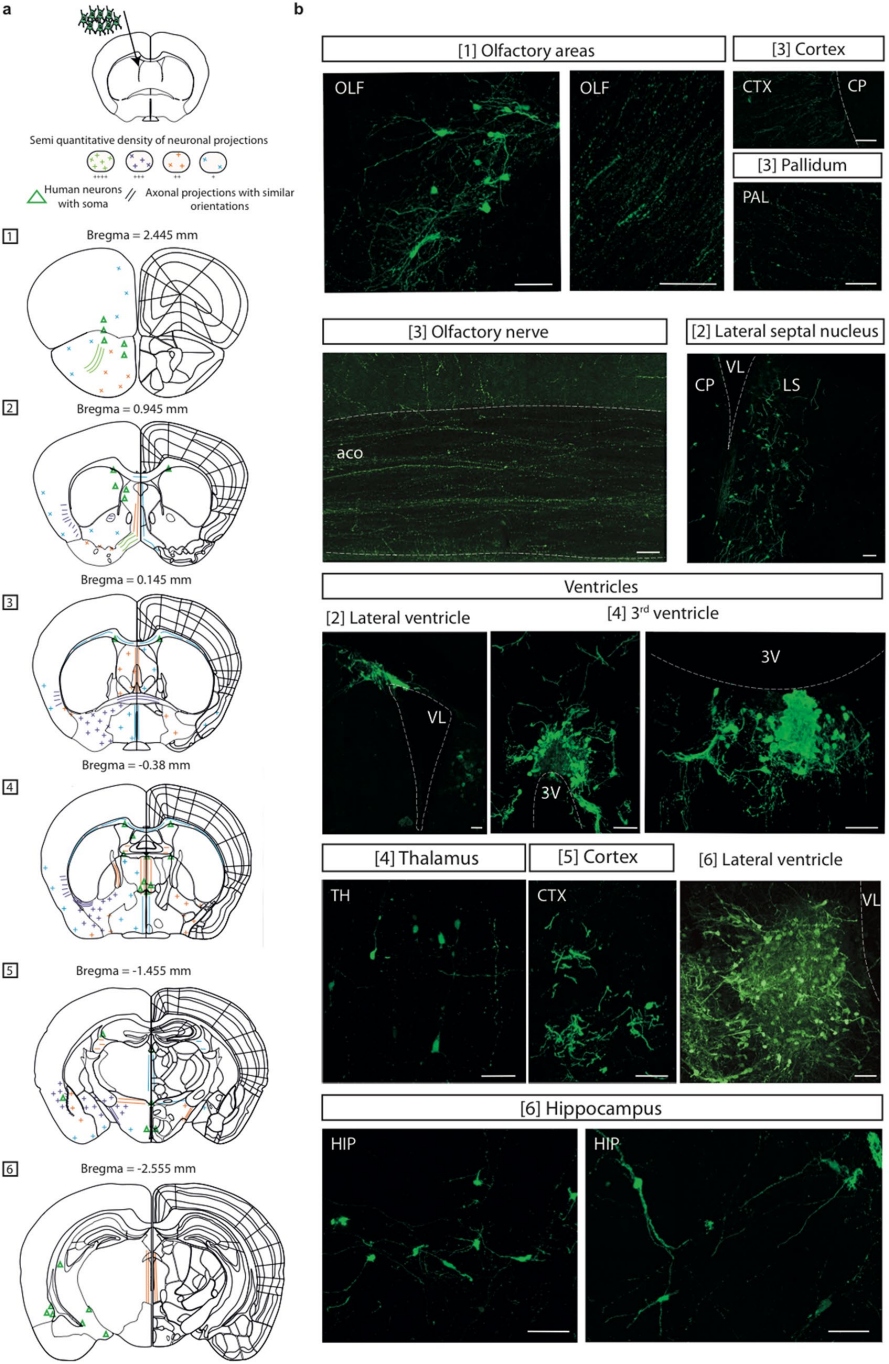
Integration of human iPSC-derived neurons into mouse brain ventricles and maturation pattern of axonal projections. (**a**) Camera lucida drawings of the pattern of axonal projections 50 days after transplantation within mouse ventricles. Abbreviations are according to Allen Brain Atlas (https://mouse.brain-map.org/). Image credit: Allen Institute. Neuron location, migration and densities are depicted using a code-color schematic representation. Results are from two independent mice, using 72 brain slices of 50 μm each. The illustrated data are from one representative brain. (**b**) Visualization of GFP-positive cells with their cell bodies, dendrites and axons (green color) within the ipsilateral ventricle. Structures from the olfactory system (Bregma 2.445 mm; 0.145 mm), lateral septal nucleus (Bregma 0.945 mm), a medial olfactory area, bed nuclei of the stria terminalis (Bregma 0.145 mm), a relay center composed of high densities of fibers, and piriform cortex (Bregma − 0.38mm), receiving back projections from olfactory bulb. Data illustrate the integration of human neurons in structures situated on the outside walls of the lateral ventricles, as well as their possible migration within the rostral migratory stream for integration within olfactory regions. No labeling was observed along the subgranular neurogenic zone of the dentate gyrus. A faint labeling was observed in corpus callosum and in some contralateral lateral areas (**a**). Scale bars = 50 μm. (aco) Anterior commissure, olfactory limb; (CP) Caudate putamen; (CTX) Cortex; (HIP) Hippocampal region; (OLF) Olfactory areas; (PAL) Pallidum; (VL) Lateral Ventricle; (3V) Third Ventricle (LS) Lateral septal nucleus; (Ipsi) Ipsilateral; (Contra) Contralateral.

**Figure 3:**
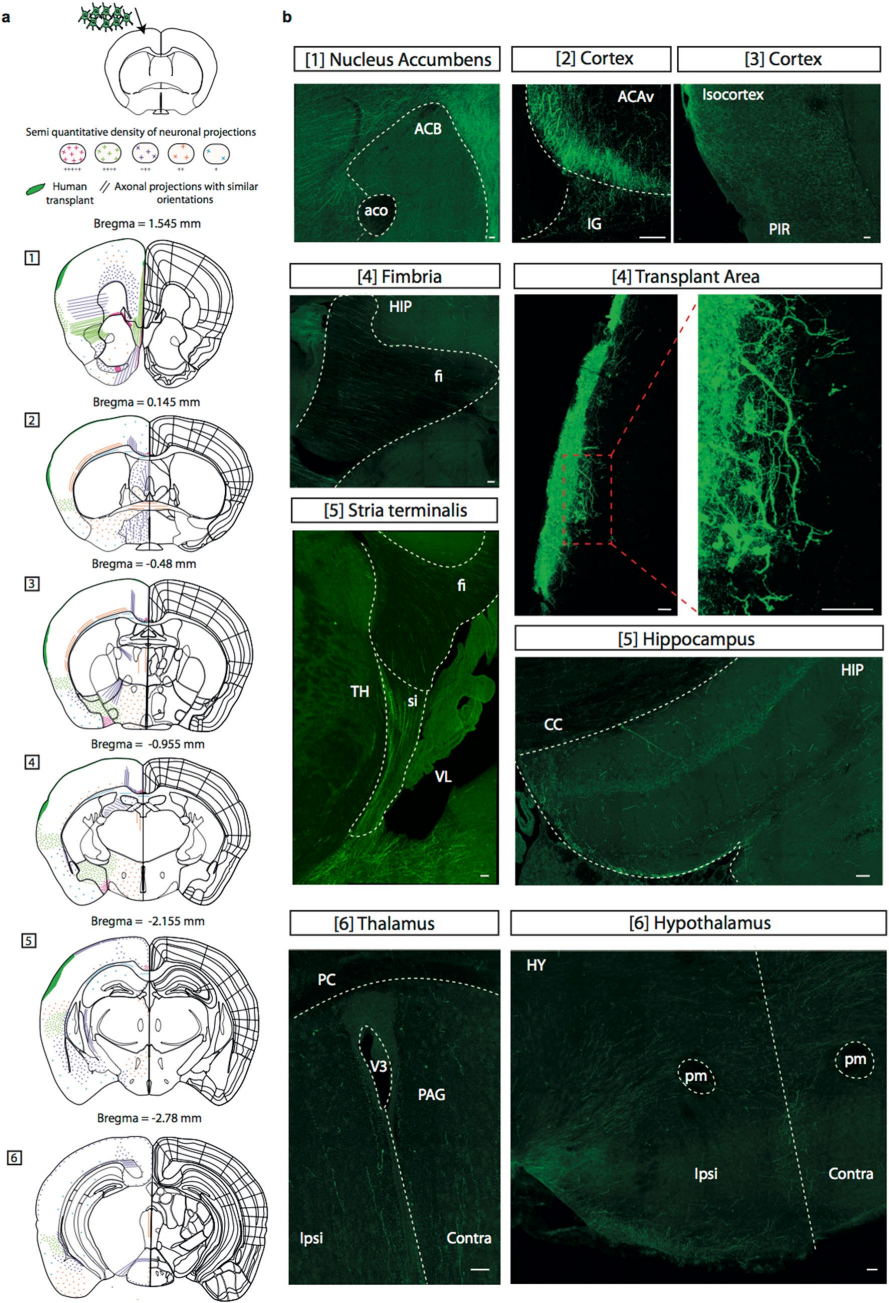
Integration of Human iPSC-derived neurons into mouse brain frontal cortex and maturation pattern of axonal projections. (**a**) Camera lucida drawings of the pattern of axonal projections 50 days after transplantation within mouse brain frontal cortex. Abbreviations are according to Allen Brain Atlas (https://mouse.brain-map.org/). Image credit: Allen Institute. Neuron location, migration and densities are depicted using a code-color schematic representation. Results are from two independent mice, using 65 brain slices of 50 μm each. The illustrated data are from one representative brain. (**b**) Visualization of GFP-positive cells with their cell bodies, dendrites and axons (green color) within the ipsilateral regions. Axons are found mostly in cortical areas: motor and somatosensory cortical layers, anterior cingulate area (Bregma 1.545 mm), nucleus accumbens, claustrum and endopiriform nucleus (Bregma 1.545 mm), Fimbria (Bregma − 0.955 mm) and to a lesser extent in hippocampus, thalamus and hypothalamus (Bregma − 2.155 mm). No labeling was observed in substantia nigra (Bregma − 2.78 mm). A labeling was observed in corpus callosum and in some contralateral lateral areas (**a**). Scale bars = 50 μm. (ACAv) Anterior cingulate area ventral part; (aco) Anterior commissure, olfactory limb; (ACB) Nucleus accumbens; (CC) corpus callosum; (HIP) Hippocampal region; (IG) Induseum griseum (supra-callosum gyrus); (Fi) Fimbria; (HY) Hypothalamus; (PIR) Piriform area; (pm) Principal mamillary tract; (PAG) Periaqueductal gray; (V3) Third ventricle; (VL) Lateral ventricle; (PC) Posterior commissure; (TH) Thalamus; (si) Substantia innominata; (Ipsi) Ipsilateral; (Contra) Contralateral.

**Figure 4:**
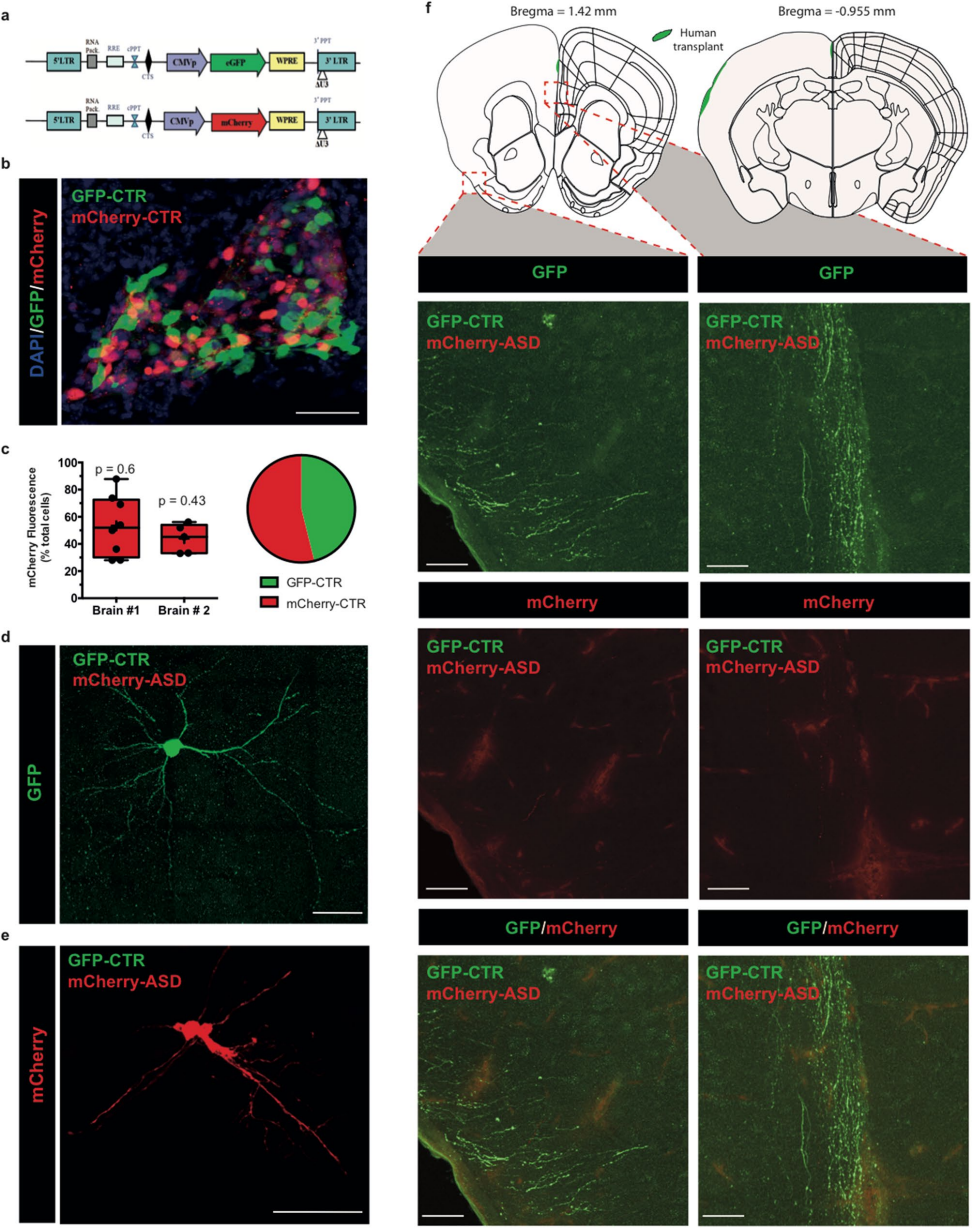
Co-transplantation of control and mutated human NPC within newborn mouse cortex. (**a**) Schematic representation of lentiviral constructs for transduction of NPC with either the GFP (green fluorescence) or the mCherry (red fluorescence) proteins using a human cytomegalovirus promoter (CMVp). (**b**) Comparative distribution of mCherry and GFP labeled CTR cells within cortical transplant. (**c**) Left: Comparative fluorescence proportions of mCherry and GFP labeled CTR cells among several graft sub areas (the number of values is indicated in the graph). Data are presented using a Box and whiskers graph with plotted “min” and “max” and median values. Mean values are also indicated as “+”. Statistical analysis was performed using a Wilcoxon signed rank test to compare medians to the theorical value “50”. *p* values are indicated in the graph. Right: Graphic camembert illustrating the percentages of mCherry-labeled control cells and of GFP-labeled control cells. Similar data were obtained between 2 transplants from 30-day old mice. (**d**, **e**) confocal images of isolated human pyramidal neurons after transplantation of CTR (green) and ASD (red) NPC from 30-day old mice. (**f**) Representative axonal projections of human GFP-labeled CTR neurons and mCherry-labeled ASD neurons in the piriform cortex (left panel) and in the infralimbic cortex (right panel) at 30 days after transplantation. Image credit: Allen Institute. Scale bar = 50 μm.

The detailed mapping of axonal projections from ventricular transplants is presented in Fig. 2. A semi-quantitative analysis indicates the existence of variable densities of GFP-positive axons within distinct brain areas. The highest densities were observed within ipsilateral regions as compared with contralateral regions. GFP-positive axons were also detected within the brain structures close to the external walls of lateral and third ventricles. This suggests a possible migration of labeled cells within the rostral migratory stream and their integration into distinct olfactory regions comprising the olfactory nerve, olfactory areas, lateral septal nucleus, and piriform cortex also named “olfactory cortex”. GFP-positive axons were also detected in cortex and the subcortical areas including hippocampus, and thalamus (Fig. 2a, b). The detailed mapping of axonal projections from cortical transplants is presented in Fig. 3a. Most of the transplants were located underneath or within the frontal cortex and did not result in a major increase of the cortical surface. Most of the GFP-positive cells remained confined within the transplant. The pattern of axonal projections was different from those observed under ventricular transplantation. Projections arising from different cortical layers were found in thalamus, hypothalamus, striatum (nucleus accumbens), ipsilateral and to a lesser extent in contralateral cortices (Fig. 3a, b). GFP-positive fibers were observed in the stria terminalis (Fig. 3a, b). Our results show an enrichment of axonal projections within the fimbria region with axonal projections which may arise from hippocampus and medial septum (Fig. 3a, b). Single GFP-neurons also settled outside the grafts, with a typical pyramidal morphology (Figs. 2b, 3b, 4d). By immunostaining methods, we were able to detect the expression of the cortical markers Cux1, Cux2 and Brn2 (Supplementary Fig. 2). We also detected individual SOM interneurons that colocalized with GFP. No colocalisation of positive parvalbumin (PV) neurons with GFP-expressing cells was observed (Supplementary Fig. 2). The expression of MAP2 and VGlut-1 proteins is depicted in Supplementary Fig. 3. These data confirm that transplanted NPC can give rise to pyramidal cells at later stages of maturation, as we described previously^23^.

### Co-transplantation of GFP- and mCherry-labeled NPC and neuronal phenotypic analysis at early stages of maturation

**Figure 5:**
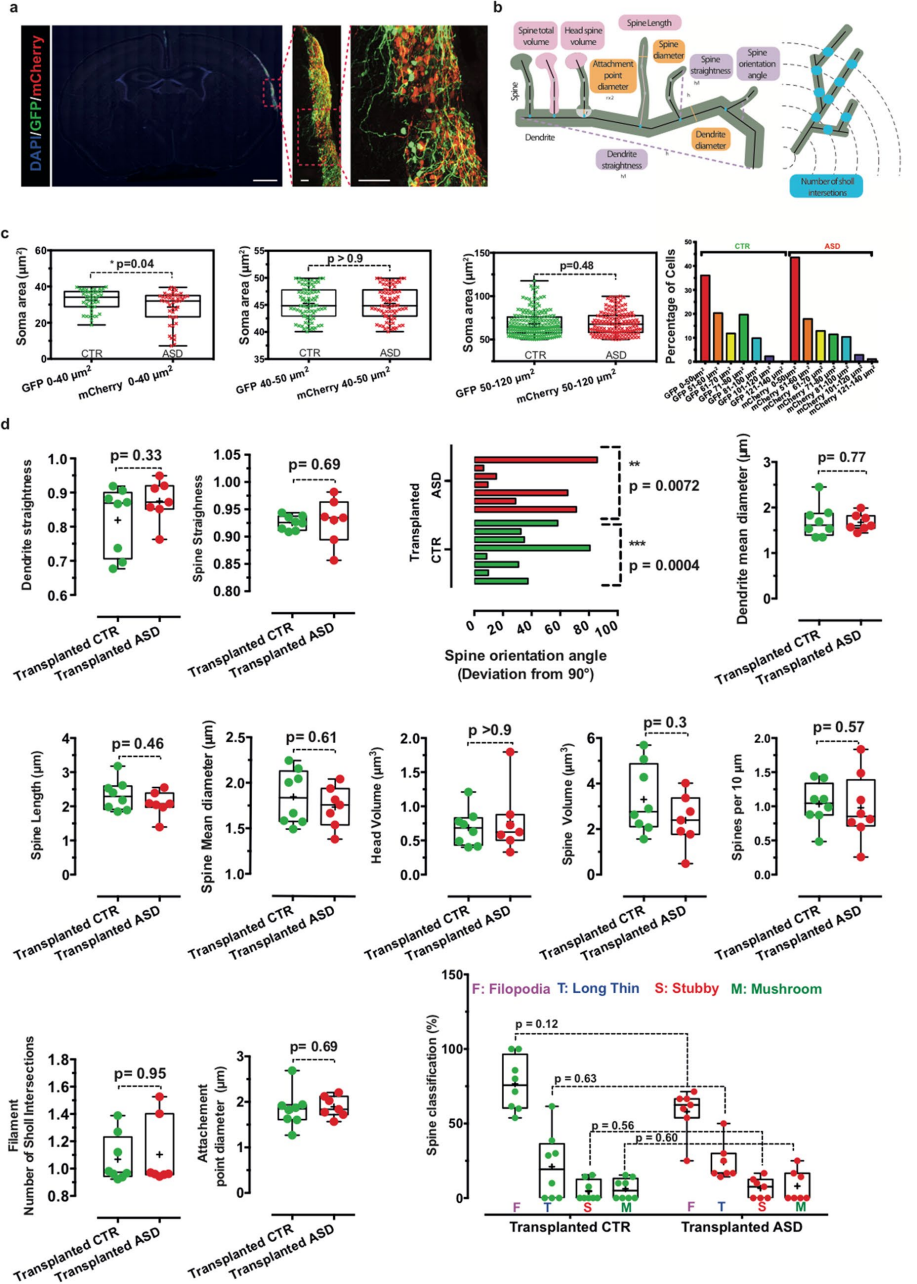
Quantitative analysis of morphological parameters of dendrites and dendritic spines from the neurons of transplanted control individual and ASD patient NPC. (**a**) Confocal image of a cortical transplant at different magnification. Scale bar = 100 μm (left panel), Scale bar = 50 μm (middle and right panels). (**b**) Schematic representations of each parameter evaluated using Imaris 9.2 software. (**c**) Classification and measurement of soma areas. (**d**) Measurement of morphological parameters of dendrites and dendritic spines. Dendrite segments are endowed with four categories of spines classified as: Filopodia (pink color), Long Thin (blue color), Stubby (red color) and Mushroom (green color). Number of neuronal dendrites is indicated in the graph. Data are presented using a Box and whiskers graph with plotted “min” and “max” and median values. Mean values are also indicated as “+”. Statistical analysis was performed using a Mann–Whitney test. For the measurement of spine deviation angle, the statistical analysis was performed using a Wilcoxon signed rank test to compare medians to the theorical value “90”. In all cases, *p* values are directly indicated in the graph. ***p < 0.001; **p < 0.01.

We co-transplanted human GFP-CTR cells either with mCherry-CTR cells or with mCherry-ASD cells. Labeled NPC were initially purified by FACS sorting in order to obtain equivalent levels of fluorescence in NPC transduced either with a lentivirus expressing GFP or a lentivirus expressing mCherry as detailed in Fig. 4a–c. Under these conditions, equivalent fluorescence levels were quantified within cortical transplants (Fig. 4b, c) as also illustrated in pyramidal neurons from 30-day old mice (Fig. 4d, e). When GFP-CTR and mCherry-ASD cells were co-transplanted, we observed a large decrease in the densities of axonal projections arising from ASD cortical neurons as compared to CTR cortical neurons (Fig. 4f). We did not visualize any cortical projection in mice with smaller and irregular transplants (data not shown). We then measured the neuronal soma size of human grafted CTR and ASD neurons within mouse cortex (Fig. 5a, c). Our results show a reduction in soma areas in grafted ASD neurons as compared to CTR neurons. This phenotype was specifically observed for somas with areas that were smaller than 40 µm^2^ (Fig. 5c). We did not observe any difference in cell morphology between human ASD cells grafted either alone or with human control cells (Supplementary Fig. 4). Caspase-3 immunostaining did not reveal any difference between transplanted CTR and ASD cell populations (Supplementary Fig. 4). In addition, we did not observe any apparent structural disaggregation with a well-defined focal nuclear labeling within ASD human cells (Supplementary Fig. 4). Finally, we also quantified diverse dendrite and spine parameters within transplants using the Imaris Software (Fig. 5b). Our results did not reveal any significant difference in morphological parameters of dendrites and spines from transplanted CTR and ASD neurons at 30 days post-transplantation (Fig. 5d).

## Discussion

We recently published an in vitro characterization of iPSC-derived neurons from control individuals and patients with ASD with single heterozygous de novo mutations in SHANK3, which is a scaffolding protein specifically expressed at synapses of excitatory neurons^1^. Based on previously published methods, we were able to derive human iPSC into neurons which consisted of up to 80% pyramidal cells, expressing selective markers of supra-granular cortical layers II–IV^1,13,16^. Human neurons developed different dendritic spine categories after 6–7 weeks in culture, however, the spine densities were decreased as a function of *SHANK3* expression^1^. The recent generation of humanized chimeric mouse brains, repopulated by human neurons^20^ offers new possibilities to study neuron phenotypes in vivo. Our first objective in the present study was to test this approach in the context of ASD, SHANK3, and pyramidal neurons. In a first series of experiments, we analyzed the integration and maturation of grafted control NPC within either the ventricles or the motor cortex of newborn mice. We showed that the transplanted cells expressed Cux1/Cux2 and Brn2 markers corresponding to cortical layers II-IV as observed in in vitro studies^16^. We also detected a few somatostatin-expressing neurons that are one of the two major classes of inhibitory interneurons in the human neocortex and hippocampus^24^. However, in the present study we did not detect PV neurons that represent the other main class of inhibitory neocortical cells. This indicates that under our experimental conditions, PV neurons were not derived from NPC, and this is in accordance with the recent results reported by D’Alessio et al.^23^. We compared the spinogenesis in human pyramidal neurons which had been previously kept in culture for 40–50 days^1^^,^ with spinogenesis of pyramidal neurons derived from transplanted NPC in newborn mice 50 days after transplantation. We observed a significant decrease in spine densities in transplanted pyramidal cells under in vivo conditions as compared to those which were kept in culture. By contrast, spine straightness and lengths were significantly increased in these in vivo studies as compared with those in in vitro conditions. The spine categories were also slightly different with an increase in the percentage of immature spines such as filopodia. We also observed a significant decrease in the percentage of stubby spines, indicating some defects in spine maturation. Our data indicate that under in vivo conditions, the maturation of grafted human neurons takes longer than they do under cultured in vitro conditions. A higher dendritic spine straightness may result from increased actin dynamics and developmental plasticity within a 3D cell environment^25^. To our knowledge, there has not been any comparative study on spinogenesis so far. It should be noted that the initial in vitro differentiation steps which were carried out before transplantation in previous studies^20,26^ may have modified the outcome of maturation processes of grafted human precursors at NPC stages.

In order to characterize our method further, we analyzed the axonal projections. We did not observe any graft boundaries initially, suggesting a full integration of human cells within the host brain. For ventricular injection, we showed that the GFP-positive human cells could migrate from the ventricular zone, project their axons towards the cortical target regions and along the rostral migratory stream towards the olfactory bulbs (Fig. 2). For cortical injection, the axon elongation was targeted towards more specific ipsilateral structures including motor cortex, striatum, thalamus, hypothalamus and hippocampus. The pattern of axonal projections was similar to that observed by other authors using human ESC-derived neurons^20^. Our data show that axonal projections may arise from distinct cortical layers. However, under our experimental conditions and at this stage of analysis, a less intense labeling was observed within the contralateral cortex. This may be due to the presence of low density of fibers within corpus callosum (Fig. 3), a structure connecting the two hemispheres.

The second objective of the present study was to take a further step towards understanding the consequences that are associated with *SHANK3* mutations. Recent reports by Schafer et al. indicate that the events which occur during early brain development, specifically from the stage of NPC, may contribute to the pathogenesis of ASD^19^. Since *SHANK3* is expressed in the human fetal brain as early as 35 weeks post-conception (BrainSpan, https://www.brainspan.org/rnaseq/search/index.html), it is reasonable to assume that in addition to its synaptic role, it may play other earlier functional roles after its cellular expression. Schafer et al. have shown an aberrant neurodevelopmental growth dynamic in maturing neurons derived from the iPSC of several patients with ASD, triggered by an altered chromatin accessibility^19^. In a second set of experiments, we analyzed the role of a *SHANK3* mutation in vivo to determine if transplanted neuronal precursors mutated in *SHANK3* respond to endogenous guidance cues from host brain, and if they behave differently as they do in vitro in culture. We selected a patient with severe autistic traits from our previous study, who presented a frameshift mutation in *SHANK3* which leads to a premature STOP codon^1^. For an accurate comparison of phenotypes within cortical transplants, we co-transplanted NPC derived from a CTR individual and those from the patient with ASD. We had initially checked for identical levels of fluorescence between GFP-positive (CTR) and mCherry-positive (CTR) cells. We then quantified the densities of cells with either green or red fluorescence within each transplant and found no significant difference. CTR and ASD neurons displayed a mixed homogeneous distribution within the transplant with no visible boundaries with host cortical cell layers. We analyzed the development and maturation of transplants at 30 days post-injection which is reported to be a sufficient period for the development of cortical and subcortical projections^21^ and a stage at which the early events can be observed. Several morphometric studies have shown a reduction in neuronal size in the neocortex of patients with ASD^27^. We used the approach which has been developed by Lingley et al. to specifically map neuronal soma size to identify the differences between neuronal cell populations^28^. By applying the same color code and by ranging neurons within the same size categories, we were able to evaluate the distribution of the soma size of human grafted CTR and ASD neurons within the host cortex. Our results show a reduction in soma areas in grafted ASD neurons as compared to CTR neurons. This phenotype was specifically observed for somas with areas that were smaller than 40 μm^2^. In agreement with our results, other authors have recently reported a reduction in cell soma size in cultured iPSC-derived neurons under *SHANK3* knockdown conditions and during the early stages of maturation^29,30^. We did not observe any difference in cell morphology between human ASD cells transplanted alone or with the control cells. This indicates that the presence of CTR cells does not modify the survival of ASD cells under our experimental conditions. These data are in accordance with those we obtained using separate cultures of CTR and ASD cells^1^. We did not detect any increase in caspase-3 labeling in ASD neurons as compared with CTR neurons. These data suggest that at early stages of neuronal development and maturation, the reduction in cell soma size is neither a consequence of apoptotic events with neuronal death, nor a consequence of the existence of deleterious experimental conditions. In addition, it has been reported that vascularization of transplanted human cerebral organoids reduces cell apoptosis^31^. We have also reported a strong vascularization of transplants from NPC by two-photon imaging^23^. This suggests that vascularization may have a strong protective effect as well. We also observed a decrease in the densities of cortical axonal projections from ASD neurons as compared to CTR neurons, as illustrated for the infra-limbic and piriform cortical areas. Our data are in agreement with those of Pagani et al. who recently reported a defective short-range cortico-cortical wiring in a mouse model presenting a homozygous loss of the Shank3B isoform^17^. In contrast to our previous in vitro results, we did not observe any significant difference in morphological parameters of dendrites and spines from transplanted CTR and ASD neurons. Since SHANK3 is known to play a crucial role in both synaptic development and function, our results suggest that compensatory changes may occur under in vivo conditions, as was recently observed in Shank3 knock-out mice having partial deletions of exons^32^. Other authors also reported unexpected levels of complexity and modularity of SHANK3 functions in vivo using mouse models^33^. Our findings confirm that a heterozygous mutation in *SHANK3* associated with a reduced *SHANK3* expression^1^ may significantly dysregulate some initial properties of neuronal development and maturation, at least with respect to cortical neurons. The fact that we did not observe any modification in morphological parameters of dendritic spines under in vivo situations as compared to in vitro models, supports the hypothesis of diverse layers of complexity in the function of SHANK3, as well as its isoforms and the other members of SHANK family. These observations are also in line with the diversity of phenotypes, including the lack of behavioral effects that are observed in heterozygous Shank3 mouse models which have been developed^34,35^.

In conclusion, as compared to in vitro studies, the humanized chimeric mouse models offer the possibility to better understand the role of human mutations that are associated with ASD and provide the opportunity to visualize the neuronal phenotypes in more details.

## Methods

We confirm that all methods were carried out in accordance with relevant guidelines and regulations. We also confirm that the experimental protocols were approved by the named institutions. Informed consents were obtained from all subjects as detailed in our previous study^1^.

### Production of human pluripotent stem cells and their derivation into NPC

The production of iPSC, their characterization, commitment to the neural lineage and derivation of stable NPC were performed according to Boissart et al.^16^. For the control iPSC lines, we used GM04603 and GM01869 from Coriell Biorepository (Coriell Institute for Medical Research, Camden, NJ, USA). We also used the line PB12, which we described in our previous study. The NPC obtained from the three control individuals for this study were referred to as “C1 + C2 + C3” in the present study. Briefly, 6-well culture plates with glass coverslips were coated under the flow hood with 0.01% poly-ornithine solution diluted to 1/6 in DPBS. After an overnight exposure at 37 °C, the coverslips were washed three times with DPBS. Then we added the laminin stock solution of 1 mg/ml, which was diluted 500 times in DPBS and incubated the coverslips for at least 4 h. at 37 °C. The NPC were then plated at the density of 50,000 cells/cm^2^ in the 6-well culture plates with the glass coverslips in 3 ml of culture medium. The culture medium consisted of 500 ml of DMEM/F-12 mixed with 500 ml of Neurobasal medium and supplemented with 2 vials (5 ml each) of N2 supplement, 2 vials (10 ml each) of B27 supplement, 10 ml of Penicillin-Streptomycin (Penicillin = 10,000 units/ml and Streptomycin = 10,000 units/ml), 1 ml of 50 mM 2-mercaptoethanol Stock solution and laminin (1/500), as described previously^1^. After removal of culture medium, fresh N2B27 medium containing 2 μg/ml of fresh laminin solution was added to keep the neurons attached on the glass coverslips and to avoid clumping. Medium was changed every 3 days.

For the in vivo transplantation into newborn mice, we used NPC derived from the GM04603 control individual (CTR) and from the “AUN291_3/L1142Vfs*153” ASD patient as representative samples from our previous study^1^. The patient for this study carried a de novo frameshift mutation which leads to a premature STOP codon. The pedigree of the family carrying the L1142Vfs*153 mutation as well as the clinical features of the patient are presented elsewhere^1,3^.

### Lentiviral transduction

NPC were labeled either by transduction with a CMV-GFP-lentivirus (green fluorescence) or with a CMV-mCherry-lentivirus (red fluorescence) as described in Gouder et al.^1^. Lentiviral vectors were prepared according to a published protocol^36^. The map of the CMV vector is detailed in Fig. 4a. NPC were transduced with 99.5 ng of lentivirus in a 6-well culture plate filled with 500 µl of fresh culture medium for 3 h. The volume was completed to 2 ml and incubated for the next 48 h, and then rinsed with culture medium. For co-transplantation, transduced NPC were initially purified by FACS in order to inject equivalent levels of green or red fluorescence intensities within cells. Labeled NPC were amplified using the same culture medium described above supplemented with brain-derived neurotrophic factor (20 × 10^−3^ μg/ml), epidermal growth factor (10 × 10^−3^ μg/ml) and fibroblast growth factor (10 × 10^−3^ μg/ml), then aliquoted in cryotubes using FBS with DMSO (10%) and frozen in liquid nitrogen. Eight to 10 days before transplantation, labeled NPC were thawed, plated and amplified using the same supplemented culture medium described above. Culture steps of NPC are detailed in Figure S1. Products and suppliers are listed in Table S1.

### Intracerebral transplantation

Cultured NPC were dissociated and resuspended in DPBS (10^5^ cells/µl) before transplantation according to D’Alessio et al.^23^. GFP-labeled cells were transplanted into either the motor cortex or into the lateral ventricle of immunodeficient NOD/SCID mice (Charles Rivers Laboratory), at postnatal day 1–day 2. Pups were first locally anesthetized using a lidocaine gel. The head of newborn pups was gently held horizontally between the operator’s fingers. Bregma was used as a visible landmark to localize the injection site. We used the published coordinates Dorso-Lateral = + 1 mm, Antero-Posterior = − 1 mm, depth = − 1 mm to target the cortex, or depth = − 2 mm to target the ventricle^23^. Two µl of resuspended cells were injected slowly using a Hamilton syringe (Hamilton, 65460) unilaterally. The syringe needle was set for injecting at 1- or 2-mm depth and was kept in the pups’ brain for 30 s following the injection before it was removed. The injection itself was done over 1 minute. This protocol was approved by the Institut Pasteur Ethics Committee and the French “Ministère de l’Education Nationale, de la Recherche et de l’Innovation” under reference APAFiS #20686. The accuracy of injection was monitored by immunofluorescence labeling of GFP-labeled transplants, after brain slicing at different periods post-injection. All pups per litter were injected and their survival was evaluated at 60%. Only the mice which presented well-defined GFP-labeled transplants within the same cortical areas were kept for further analysis. The co-transplantation of equal numbers of CTR and ASD cells was monitored by initial cell counting and immunofluorescence labeling of the distinct cell populations. Brains with small number of labeled transplanted cells were excluded from our analysis, even though they presented similar phenotypes.

### Immunofluorescence

Immunofluorescence analysis on brain sections was performed at 30 and 50-days post-transplantation according to our previously described protocol with slight modifications^1,37^. Animals received a lethal dose of a mixture of Ketamine (Imalgen®, 100 mg/kg, Merial) and Xylazine (Rompun®, 10 mg/kg, Bayer), and were transcardially perfused with 15 ml of DPBS, followed by 50 ml ice-cold paraformaldehyde (PFA 4%) in 0.1 M phosphate buffer (pH 7.4). Brains were dissected and post-fixed overnight at 4 °C in 4% PFA. Fifty μm coronal sections were obtained using a vibratome (Leica Biosystems, VT1000S), collected in 24-well-culture plates, and stored in DPBS at 4 °C until use. Briefly, the free-floating sections were rinsed twice in PBS (pH 7.4) and then incubated in DPBS solution containing 0.5% Triton X-100 and 10% serum at room temperature (RT) for 30 min to block nonspecific binding. Primary antibodies, diluted in blocking solution, were added overnight at 4 °C. The list of primary and secondary antibodies with their respective dilutions is presented in Table S2. In order to improve the labeling of the whole spine morphology, immunofluorescence labeling was performed using either an anti-GFP antibody or an anti-mCherry antibody in permeabilized conditions. An additional incubation with DAPI diluted in DPBS was performed for 10 min at room temperature.

### Image quantitative analysis

We have previously reported a stepwise protocol for imaging of dendritic spines after immunofluorescence in a video journal^37^. Briefly, confocal imaging was performed using a confocal laser-scanning microscope with a 40X oil NA = 1.3 objective, a 488 nm laser for GFP excitation, and a 555 nm laser for mCherry excitation. A Z spacing ranging from 150 to 300 nm was used for Z-stack acquisitions. A semi-automatic tracing of dendrites and automated segmentation of spines was performed using the Filament Tracer module of Imaris 9.5 software (Bitplane AG, Zürich). Spine categories were defined by their morphology as follows: Stubby: length < 1 μm; Mushroom: Length (spine) < 3 μm and Max width (head) > mean width (neck) × 2; Long thin: Mean width (head) ≥ Mean width (neck); Filopodia-like: length ≤ 4 μm (no head). For the quantification of cell soma areas of transplanted cells, Z-stacks were acquired under the same microscopic parameters. Soma size areas were measured using Fiji software.

### Statistics

Statistical analysis was performed using GraphPad Prism Version 6 software (GraphPad, San Diego, California, USA). Statistical analyses are reported in each figure legend. 95% confidence levels have been used.

## Supplementary information

Supplementary Information.

## Data Availability

All data generated or analyzed during this study are included in the published article (and its Supplementary Information file).
