# Supplementary material for: A chimeric mouse model to study human iPSC-derived neurons: the case of a truncating SHANK3 mutation
Source: Sci Rep. 2020 Aug 7;10:13315. doi: 10.1038/s41598-020-70056-4 (PMC7414912; doi:10.1038/s41598-020-70056-4)
Supplement: Supplementary file 1 — Supplementary Information. [file 41598_2020_70056_MOESM1_ESM.pdf]

A chimeric mouse model to study human iPSC-derived neurons: the case of a truncating *SHANK3* mutation

Aline Vitrac [1], Stéphanie Pons [2], Marta Balkota [2], Nathalie Lemière [1], Célia Raïs [2, 3], Jean-Pierre Bourgeois [1], Uwe Maskos [2], Thomas Bourgeron [1] and Isabelle Cloëz-Tayarani [2] *

[1]Human Genetics and Cognitive Functions, CNRS UMR 3571 « Genes, Synapses and Cognition », Université de Paris, Institut Pasteur, Paris, France. [2]Integrative Neurobiology of Cholinergic Systems, CNRS UMR 3571 « Genes, Synapses and Cognition », Institut Pasteur, Paris, France. [3]Collège doctoral, Sorbonne Université, Paris, France.

*Correspondence and requests for materials should be addressed to I.C.-T. (email: isabelle.cloez-tayarani@pasteur.fr)

<u>Supplementary Figure 1</u>:

Culture steps for the *in vitro* and *in vivo* analysis of iPSC-derived neuronal precursors (NPC).

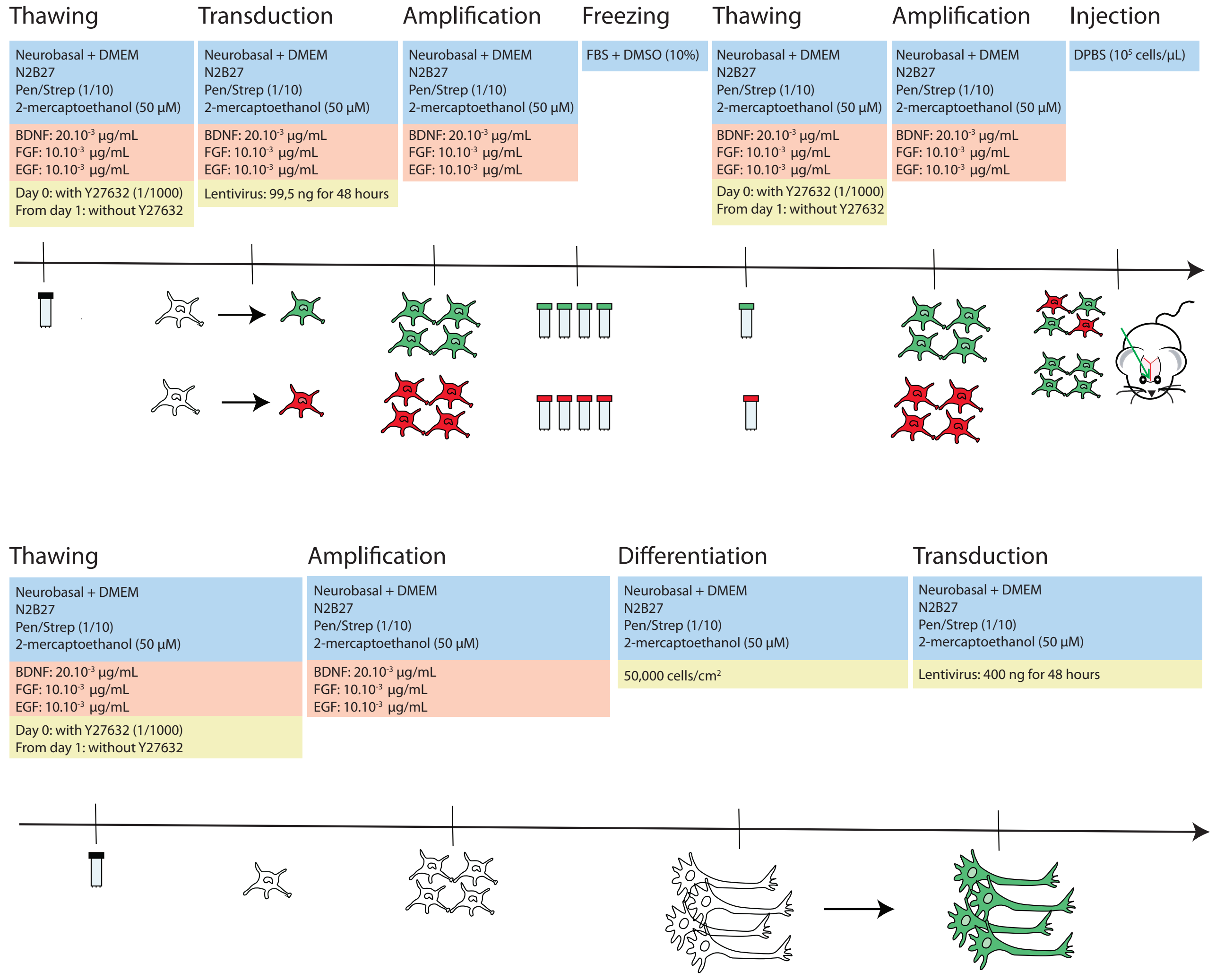

`

<u>Supplementary Figure 2</u>:

Immunofluorescence staining of human iPSC-derived neurons transplanted into the brain cortex of newborn mice, at 60-80 days post-transplantation.

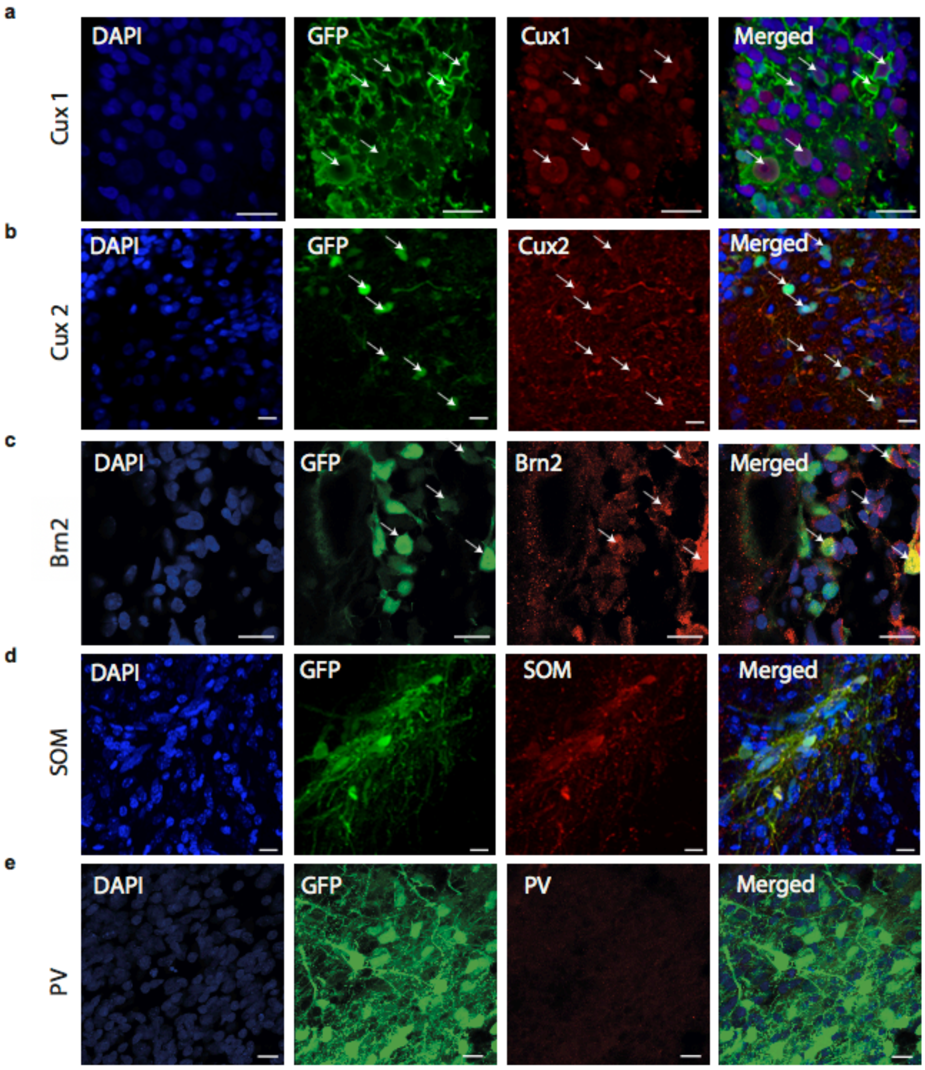

Legend to Supplementary Figure 2: Immunofluorescence staining of human iPSC-derived neurons transplanted into the brain cortex of newborn mice, at 60-80 days post-transplantation. (a-c) Immunofluorescence staining of grafted GFP-cells with Cux1, Cux2 and Brn2 antibodies, markers of the cortical layers II-IV. Cellular co-labeling of transplanted human cells which shows the expression of Cux1, Cux2 and Brn2 markers in human neuronal cells. (d-e) Immunofluorescence staining of grafted GFP-cells with Parvalbumin (PV) and Somatostatin (SOM) antibodies that are selective markers of interneurons. Cellular co-labeling shows the presence of human SOM positive cells but the human PV cells are absent. Scale bars = 15µm.

Supplementary Figure 3:

Immunofluorescence staining of human iPSC-derived neurons either kept *in vitro* or transplanted into the mouse brain cortex.

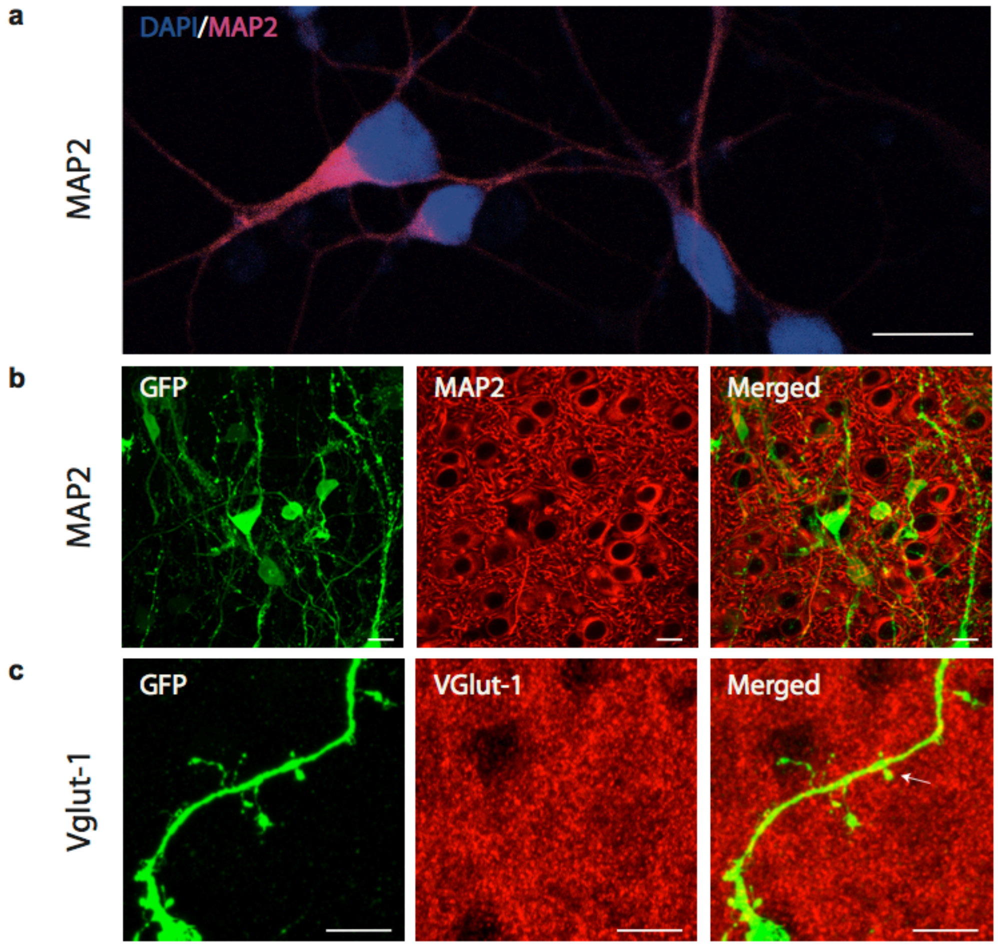

Legend to Supplementary Figure 3: Immunofluorescence staining of human iPSC-derived neurons either kept *in vitro* or transplanted into the mouse brain cortex.

(a) Immunofluorescence staining of cultured human control iPSC-derived cortical neurons by MAP2 antibody at 24 days after NPC differentiation. MAP2 antibody labels the perikaryal and dendrites of neuronal cells. (b) MAP2 staining of GFP-labeled human cells transplanted into the brain cortex of newborn mice, at 7 months post-transplantation. (c) Vglut-1 staining of GFP-labeled human cells transplanted into the brain cortex of newborn mice, at 7 months post-transplantation. Vglut-1 antibody is a selective marker of excitatory glutamatergic neurons. Target indicates the presence of human Vglut-1 positive cells within the mouse brain. Scale bars = 15μm.

Supplementary Figure 4:

Immunofluorescence staining of human iPSC-derived neurons transplanted into the brain cortex of newborn mice, at 30 days post-transplantation.

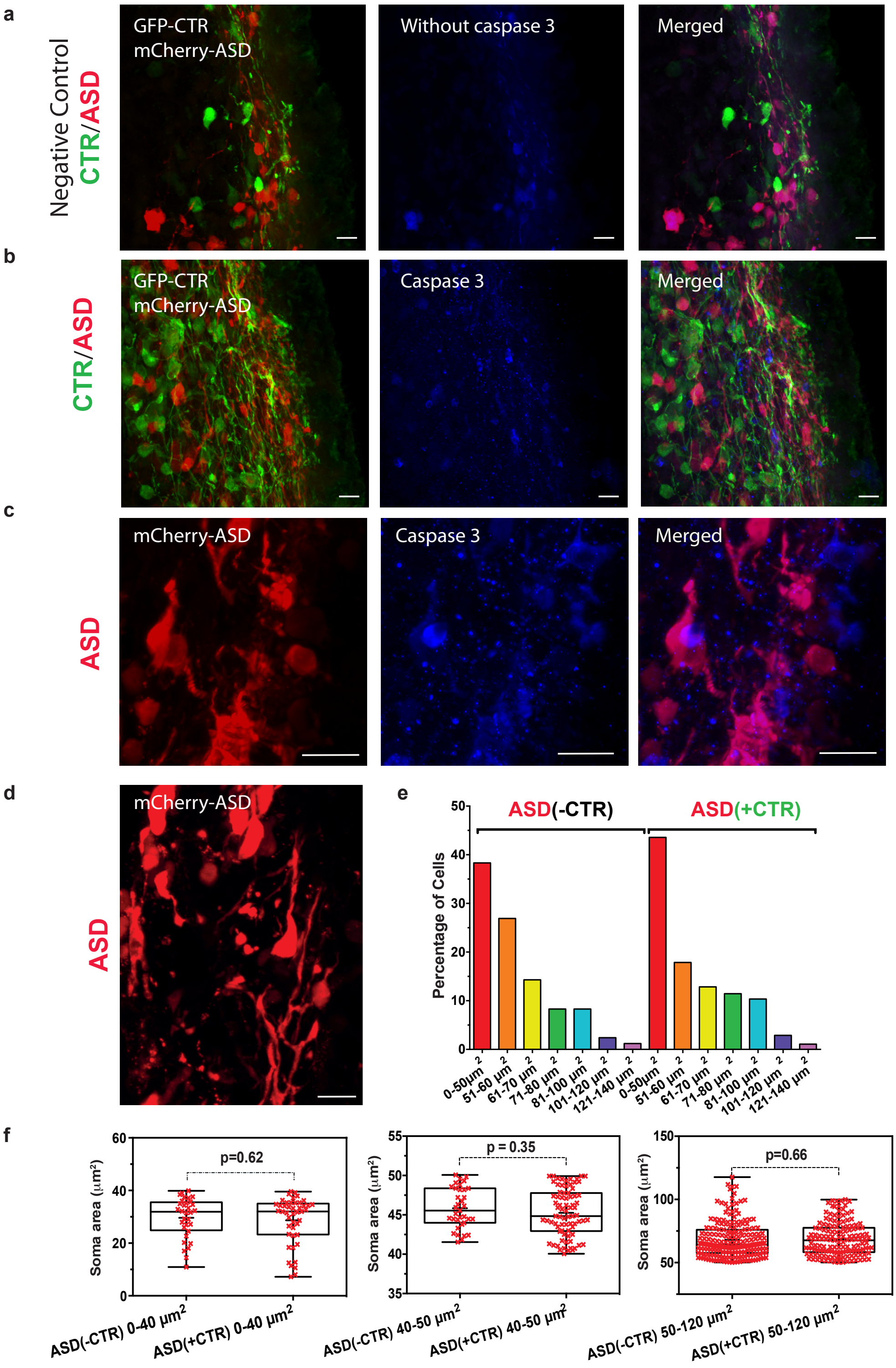

Legend to Supplementary Figure 4: Immunofluorescence staining of human iPSC-derived neurons transplanted into the brain cortex of newborn mice, at 30 days post-transplantation.

(a-b) Immunofluorescence staining of co-transplanted control GFP- and ASD mCherry cells labeled with Caspase-3 antibody, a marker of cell apoptosis. Negative control was performed in the absence of primary antibody. Despite some background staining, no well-defined focal nuclear labeling of Caspase-3 is detected within human cells (c) Immunofluorescence staining of transplanted mCherry cells labeled with Caspase-3. (d) Immunofluorescence staining of mCherry labeled human ASD neurons within a cortical transplant. (e-f) Comparative classification and measurement of soma areas between ASD cells transplanted either alone or with control cells. Statistical analysis was performed using a Mann-Whitney test. Scale bars = 15μm.

Table S1: List and references of products.

PRODUCT	REFERENCE	SUPPLIER
**Cell culture**		
Neurobasal™	21103-049	Gibco
DMEM/F-12 (1:1)+ GlutaMAX™ -I	31331-028	Gibco
B27® -Supplement (100X)	12587-010	Gibco
N2 - Supplement (50X)	17502-048	Gibco
2-Mercaptoethanol 50 mM	31350-10	Gibco
DPBS (1X)	14190-094	Gibco
Pen Strep	15140-148	Gibco
Poly L-ornithine	P4957	Sigma Aldrich
Laminine	23017-015	Invitrogen
Geltrex	A14133_02	Gibco
Y27632	04-0012-02	Stemgent
BDNF	450-02 100µg	Peprotech
EGF	AF-100-15-500 µg	Peprotech
FGF-basic	100-18b-50µg	Peprotech
DMSO	D2438	Sigma Aldrich
0,05% Trypsin-EDTA (1X)	25300-054	Gibco
FBS	P30-3306	PAN Biotech
**Immunofluorescence**		
Triton® X-100	X100 1L	Sigma Aldrich
Donkey serum	D9663	Sigma Aldrich
DAPI (1mg/ml)	62248	Thermoscienific
Prolong™ Gold antifade reagent	P36930	Invitrogen

Table S2: Commercial antibodies used for immunofluorescence labeling.

Antibodies	HOST	REFERENCE	SUPPLIER	DILUTION
**Primary Antibodies**				
GFP	Rabbit	A6455	Invitrogen	1/750
GFP	Chicken	13970	Abcam	1/2000
mCherry	Rat	M11217	Invitrogen	1/500
Cux1	Mouse	ab54583	Abcam	1/500
Cux2	Rabbit	bs-1182R	Bioss	1/50
SOM	Mouse	sc-5565	Santa Cruz Biotechnology	1/500
PV	Rabbit	PV27	Swant	1/500
MAP2	Mouse	sc-5359	Santa Cruz Biotechnology	1/200
Vglut-1	Rabbit	135 303	Synaptic System	1/500
Caspase-3	Rabbit	9661S	Cell Signaling	1/300
**Secondary Antibodies**				
Anti Rabbit Alexa 488	Donkey	A21206	Invitrogen	1/200
Anti Rat Alexa 594	Donkey	A21209	Invitrogen	1/200
Anti Rabbit Alexa 594	Donkey	A21207	Invitrogen	1/200
Anti Chicken Alexa 488	Goat	A11039	Invitrogen	1/200
Anti Mouse Alexa 594	Goat	A11005	Invitrogen	1/200
Anti Rabbit Alexa 647	Goat	A21244	Invitrogen	1/200
